# Pressure-volume analysis of thermodynamic workload of voiding - an application in pelvic organ prolapse patients subjected to robotic-assisted sacrocolpopexy

**DOI:** 10.1007/s13534-024-00453-5

**Published:** 2024-12-30

**Authors:** Hui-Hsuan Lau, Cheng-Yuan Lai, Ming-Chun Hsieh, Hsien-Yu Peng, Dylan Chou, Tsung-Hsien Su, Jie-Jen Lee, Tzer-Bin Lin

**Affiliations:** 1 Division of Urogynecology, Department of Obstetrics and Gynecology, Mackay Memorial Hospital, Taipei, Taiwan; 2 Department of Surgery, Mackay Memorial Hospital, Taipei, Taiwan; 3 Department of Medicine, Mackay Medical College, New Taipei, Taiwan; 4 Institute of Biomedical Sciences, Mackay Medical College, New Taipei, Taiwan; 5 Institute of Translational Medicine and New Drug Development, College of Medicine, China Medical University, Taichung, Taiwan; 6 Department of Physiology, School of Medicine, College of Medicine, Taipei Medical University, No. 250 Wu-Shin Street, Taipei, 11031 Taiwan; 7 Graduate Institute of Biomedical Electronics and Bioinformatics, National Taiwan University, Taipei, Taiwan

**Keywords:** Pressure-volume analysis, Thermodynamics, Da Vinci Si systems, Pelvic floor reconstruction, Sacrocolpopexy

## Abstract

**Purpose:**

*Given objective benefits of robotic-assisted sacrocolpopexy (RSCP)* to the voiding function/deficit of patients with pelvic organ prolapse (POP) waits to be clarified, this study investigated if RSCP modifies voiding functions of POP patients by focusing on its impact on the outlet resistance-dependent voiding workload using pressure-volume analysis (PVA), a protocol thermodynamically assaying work expenditure by the bladder in voiding cycles.

**Methods:**

Pre- and post-operative cystometry and PVA of 22 female patients, who underwent RSCP for POP (stage ≥ II), were reviewed. *Mean voiding resistance (Rvod)*,* mean voiding pressure (Pvod)*,* mean voiding flow (Fvod)*, voided volume (Vvod), voiding time (Tvod), and the trajectory-enclosed area (Apv) were analyzed.

**Results:**

The PVA, in which trajectory shaped an enclosed loop representing a voiding cycle, was established by adapting from the time-domain cystometry. Compared to the pre-operative control, RSCP decreased Rvod, Pvod, and Tvod (*p* = 0.003, 0.042, and 0.040, respectively. All *N* = 22) but increased Fvod (*p* = 0.036, *N* = 22) without markedly affecting Vvod (*p* = 0.580, *N* = 22). Apv was decreased after RSCP (*p* = 0.017, *N* = 22). The RSCP-decreased Rvod (ΔRvod) displayed a moderate correlation with both the decreased Pvod (ΔPvod, *r* = 0.551, *p* = 0.007, *N* = 22) and the increased Fvod (ΔFvod, *r*=-0.625, *p* = 0.001, *N* = 22). The ΔFvod moderately correlated with the decreased Tvod (ΔTvod, *r*=-0.620, *p* = 0.002, *N* = 22). Moreover, the RSCP-decreased Apv (ΔApv) displayed correlation with the ΔPvod (*r* = 0.385, *p* = 0.047, *N* = 22).

**Conclusions:**

Through diminishing outlet resistance of POP patients, RSCP not only prompted urine emission thereby increased voiding efficacy but also decreased the pressure developed for driving urine flow that lessened voiding workload.

*Clinical Trial Registration* ClinicalTrials.gov (NCT05682989).

## Introduction

As the continuous advance in women’s life span, the prevalence of pelvic organ prolapse (POP), i.e., the pelvic organ protrudes beyond their confines, is growing because it commonly affects women with age elder than 70 [1, 2]. Options for symptomatic POP therapy includes consulting and observation, physiotherapy, vaginal pessary, and invasive reconstruction [3]. Among surgical interventions for POP, robotic-assisted sacrocolpopexy (RSCP), a minimal invasive procedure providing adequate apical support, has gained popularity [4, 5] because it offers excellent visualization and precise actions [6] as well as improves surgeons’ ergonomics [7] and learning curve [8, 9] compared with laparoscopic sacrocolpopexy.

Studies have provided evidence positively supporting the outcome of RSCP in POP repair by showing RSCP results in satisfactory anatomical restoration [10] and patients post-operatively report improved quality of life [9]. By focusing on the bladder function, a very recent study has demonstrated RSCP improves bladder storage by increasing compliance during bladder filling [11]. Nevertheless, the potential impact of RSCP on the bladder voiding has been scarcely investigated. Because bladder function involves urine storage and disposal [12], if RSCP modifies bladder voiding is an issue needs to be clarified.

In animal studies [13] and clinical practices [14], the well-established pressure-flow cystometry, which records intra-vesical pressure and emitted flow over time, is widely used to identify deficits of bladder functions. In addition to cystometry, the pressure-volume analysis (PVA), which inspects the relationship between bladder pressure and volume, is recently developed to assay thermodynamic [15–18] and visceroelastic [11, 19, 20] processes of micturition cycles. Particularly, in analogous to the PVA used in the cardiology [21–23], the area subtended by the trajectory loop in bladder PVA virtually characterizes the work expenditure by the bladder in a voiding cycle [16–18]. Given a lasting overload on the bladder is one of the risk factors leading to un-compensatory bladder dysfunctions [24], possible pathophysiological causes underlying deficits in voiding work as well as the potential impact of conservative and/or invasive therapy on the workload of voiding warrant clarification.

Notably, the obstruction of bladder outlet [25] caused by compression or kinking on the urethra [26] is recognized to underlie the voiding difficulties in POP patients [27]. Since obstruction of bladder outlet manifests itself as an elevated voiding resistance during emission [28] and previous publications demonstrated surgically overlaid urethral resistance increases [16] and conversely, operation-diminished urethral resistance lessens the mechanical work of voiding cycles [17, 18], in this study, we investigated if RSCP modifies voiding work to benefit the bladder function by specifically focusing on the potential impact of RSCP on the outlet resistance of bladder voiding.

## Materials and methods

### Study design

The Ethics Committee of Mackay Memorial Hospital approved this study (22MMHIS361e; 2022/12/08 Taipei, Taiwan). Protocols of this study was registered in ClinicalTrials.gov (NCT05682989). Pressure-flow studies of patients undergoing primary RSCP for POP (higher than stage II; POP-Quantification system) were reviewed. Those patients who have a history of recto-/urethra-/vesico-vaginal fistula, anti-incontinence surgery, and failed to complete followed-ups were excluded from analysis.

### Surgical procedure

POP repair was performed using a da Vinci Si Systems (Intuitive Surgical^®^, Sunnyvale, CA, USA). If necessary, a total/supracervical hysterectomy would be performed. Pelvic floor mesh (PELVI-STOP^®^, APIS Technologies, Sarl, Switzerland) was used for suspension of the vaginal vault/cervix to the sacral promontory. After dissection, the mesh’s anterior and posterior arms were respectively sutured at the anterior vaginal wall and cervix as well as the level of the levator ani and central tendon of the perineum to the cervix. After making an incision on the peritoneum and the mesh was tunneled, the mesh’s cranial part was sutured at the longitudinal ligament at the sacral promontory. Finally, re-peritonization was done to.

prevent mesh exposure.

### Cystometry investigation

Cystometry investigations were carried out complying with the guidelines of the International Continence Society [29]. Briefly, warm saline (37 °C) was instilled continuously (80 ml/min) into patient’s bladder, and the detrusor pressure (Pdet), vesical pressure (Pves), abdominal pressure (Pabd), urethral flow (Flow), infused volume (Vinf), voided volume (Vvod), and intra-vesical volume (Vive) were on-line recorded (MMS UD-200, Medical Measurement System, Enschede, Netherlands) and analyzed (Biopac MP36, Biopac Systems, Santa Barbra, US). The voiding time (Tvod; the duration of fluid emission), *mean voiding pressure (Pvod; the mean Pdet during fluid emission)*,* mean voiding flow (Fvod; the mean flow rate during fluid emission*, i.e.,* average uroflow; calculated by Vvod/Tvod)*,* and mean voiding resistance (Rvod; the mean resistance during fluid emission; calculated by Pvod/(Vvod/Tvod)) were subsequently analyzed.*

### Pressure-volume analysis

Pressure-volume analyses (PVAs) of voiding cycles were constructed by plotting Pdet against Vive [15, 16]. The trajectory-enclosed area (Apv) was assayed with a processing program (Image J, LOCI, Madison, WI, US). Though stress tests comprising cough and Valsalva maneuver obviously interfere both Pabd and Pves, publications demonstrate these tests hardly affect the Pdet [16, 30] in the cystometry and trajectory in PVA [16]. Additionally, stress tests were made during urine storage that barely affect the bladder voiding. Hence, cystometry with stress tests were included in data analysis.

### Statistical analysis

Characteristics of patients’ demographics were presented using descriptive statistics. Data were summarized as mean ± SEM. Between groups, difference in values was analyzed using paired student’s t-tests with a significant level of difference set at *p* < 0.05.

## Results

### Patients’ database

Cystometry of 22 POP patients (mean age = 62.32 ± 2.20 years old), who pre-operatively displayed prolapse ≥ stage II (POP-Q system), were reviewed and analyzed. *Excepting 1 patients displayed apical and anterior compartments prolapse*,* all patients have combined prolapse in three components before operation. Out of 22 patients*,* 3 patients displayed outlet obstruction*,* 7 patients displayed poor urinary stream*,* 12 patients displayed urinary frequency*,* 1 patient displayed urinary leakage but no patient displayed interstitial cystitis or bladder pain syndrome. Moreover*,* 3 patients have symptom of vaginal pain/bleeding*,* 13 patient have vaginal bulging/mass*,* 4 patients have bearing-down sensation.* Urodynamic evaluations were carried out at a mean of 25.00 ± 5.83 days before and 107.31 ± 11.10 days after the RSCP.

### Diminished *mean voiding resistance*

**Fig. 1:**
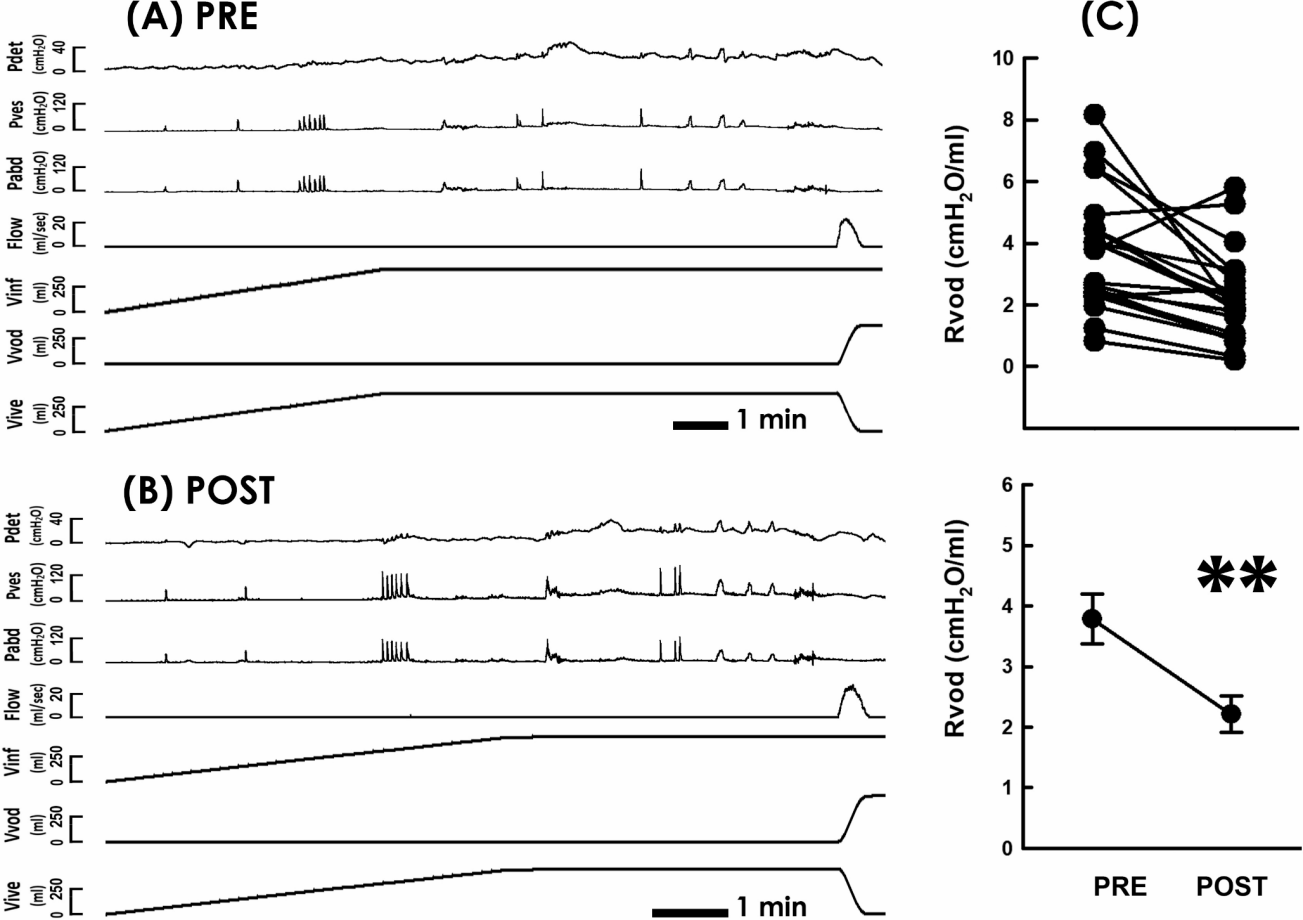
Cystometry analyses before and after RSCP. **A** and **B** Cystometry of a POP patient measured pre- and post-operatively, respectively (PRE and POST). Pdet: detrusor pressure, Pves: vesical pressure, Pabd: abdominal pressure, Flow: urethral flow, Vinf: infused volume, Vvod: voided volume, Vive: intra-vesical volume. **C** Individual (upper) and mean (lower) values of the *mean voiding resistance (Rvod)* measured pre- and post-operatively. (** *p* = 0.003 vs. PRE; *N* = 22)

Given voiding deficits in POP patients involves the outlet kinking- or compression-enhanced outlet resistance [26], we first examined if RSCP diminishes voiding resistance in POP patients. Pre- and post-operative cystometry (Fig. 1A PRE and 1B POST, respectively) demonstrated that when compared with the pre-operative control (Fig. 1C PRE), RSCP consistently decreased *mean voiding resistance (Rvod)* in most patients (19 out of 22; (19/22)) and significantly decreased the mean Rvod of the patient (*p* = 0.003 vs. PRE, *N* = 22), indicating RSCP post-operatively diminished the voiding resistance of POP patients.

### Decreased *mean voiding pressure*/Increased *mean voiding flow*

**Fig. 2:**
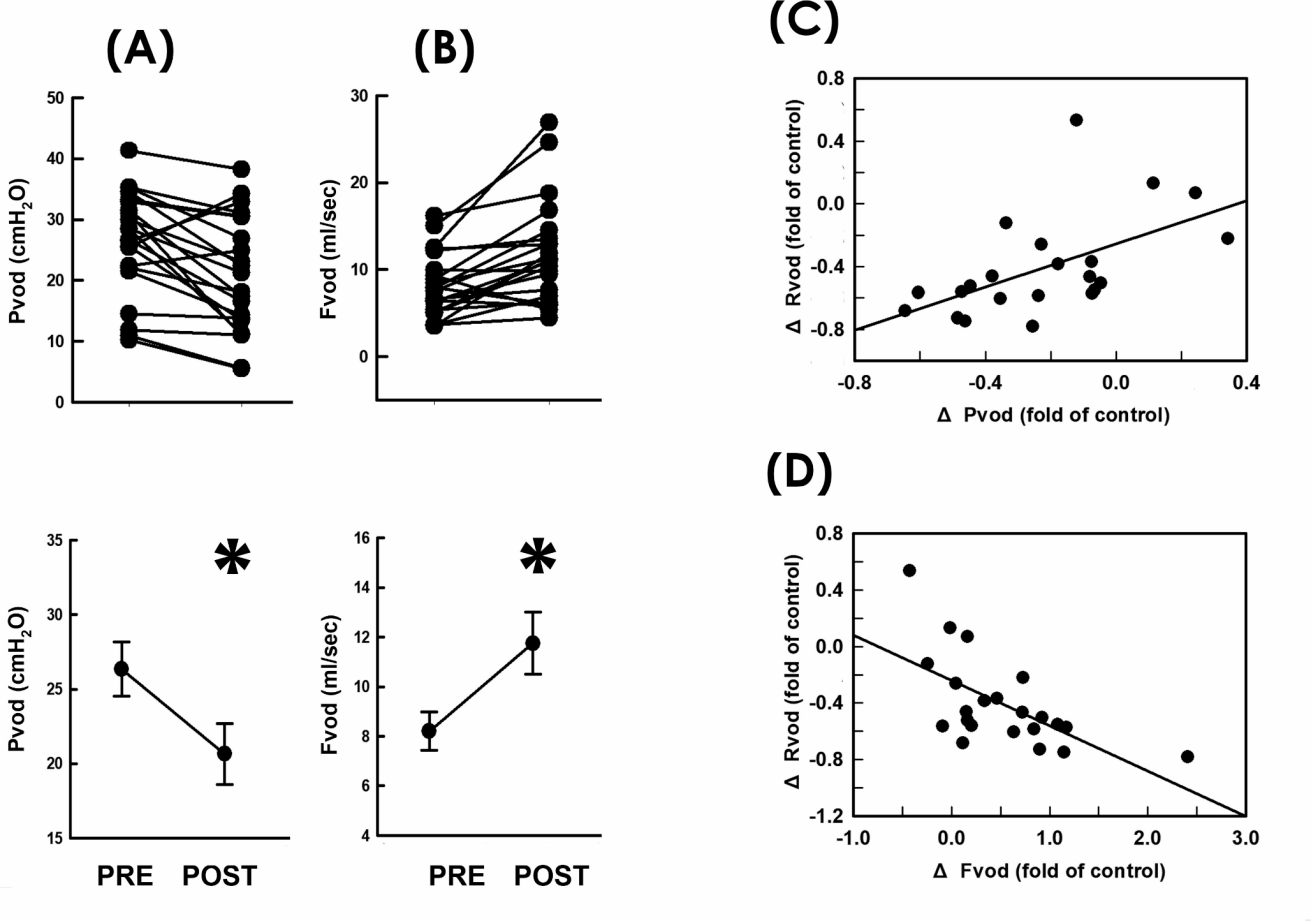
Resistance-associated parameters before and after RSCP. **A**, **B** Individual (upper) and mean (lower) values of respectively the *mean voiding pressure (Pvod*; * *p* = 0.042 vs. PRE; *N* = 22) and *mean voiding flow (Fvod*; * *p* = 0.036 vs. PRE; *N* = 22) measured pre- and post-operatively (PRE and POST). **C**, **D** Correlation analyses of the RSCP-associated change in *mean voiding resistance (ΔRvod)* with respectively the change in *mean voiding pressure (ΔPvod*; *r* = 0.551, *p* = 0.007; *N* = 22) and the change in *mean voiding flow* (ΔFvod; *r*=-0.625, *p* = 0.001; *N* = 22)

Considering the voiding resistance is calculated by dividing the voiding pressure by the flow rate, we next investigated whether the RSCP-diminished *mean voiding resistance* was associated with modified *mean voiding pressure* and/or *mean flow rate.* When compared with the pre-operative control (Fig. 2A PRE), summarized data demonstrated RSCP consistently decreased *mean voiding pressure (Pvod)* in most patients (19/22) and significantly decreased the mean Pvod of the patient (*p* = 0.042 vs. PRE, *N* = 22), indicating the pressure gradient developed for driving urine flow was decreased after RSCP. Moreover, when compared with the pre-operative control (Fig. 2B PRE), RSCP consistently increased the *mean flow rate of voiding (Fvod)* in most patients (18/22) and significantly increased the mean Fvod of the patient (*p* = 0.036 vs. PRE, *N* = 22), indicating RSCP post-operatively prompted urine emission by elevating rate of fluid emission. To confirm the causal relationships between the change of Rvod (ΔRvod) and changes of Pvod and Fvod (ΔPvod and ΔFvod, respectively) in response to RSCP, we assayed the correlation between the ΔRvod and the ΔPvod and ΔFvod. Bivariate analyses revealed the RSCP-induced ΔRvod displayed moderate correlations with both the ΔPvod (Fig. 2C, *r* = 0.551, *p* = 0.007, *N* = 22) and ΔFvod (Fig. 2D, *r*=-0.625 *p* = 0.001, *N* = 22), indicating the RSCP-diminished voiding resistance was associated with decreased ΔPvod and increased ΔFvod.

### Unmodified voiding volume/shortened voiding time

**Fig. 3:**
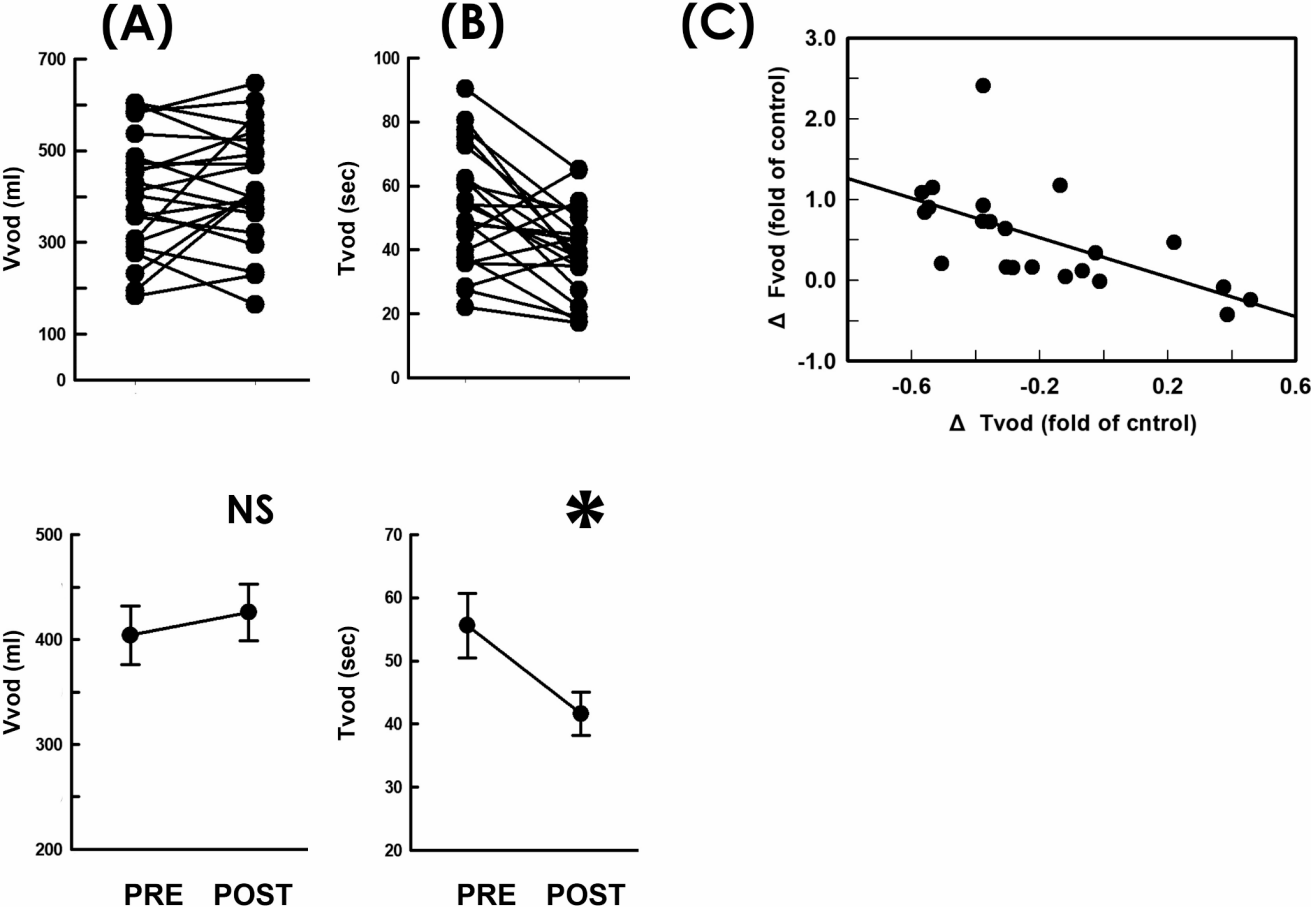
Flow-associated parameters before and after RSCP. **AB** Individual (upper) and mean (lower) values of respectively the voided volume (Vvod; NS *p* = 0.580 vs. PRE; *N* = 22) and voiding time (Tvod; * *p* = 0.040 vs. PRE; *N* = 22) measured pre- and post-operatively (PRE and POST). **C** Correlation analysis of the RSCP-associated change in voiding flow (ΔFvod) with the change in the voiding time (ΔTvod; *r* = -0.620, *p* = 0.002; *N* = 22)

Since the flow rate of voiding is calculated by dividing the voided volume (Vvod) by the voiding time (Tvod), we next assayed the impact of RSCP on the Vvod and Tvod. Compared with the pre-operative control (Fig. 3A PRE), summarized data demonstrated RSCP resulted in neither a consistent trend of Vvod change nor significantly modified the mean Vvod of the patient (*p* = 0.580 vs. PRE, *N* = 22). In contrast, RSCP consistently decreased Tvod in most patients (Fig. 3B. 18/22) and significantly decreased the mean Tvod of the patient (*p* = 0.040 vs. PRE, *N* = 22) compared with the pre-operative control. Moreover, bivariate analyses revealed the RSCP-associated Fvod change (ΔFvod) displayed a moderate correlation with the Tvod change (ΔTvod) (Fig. 3C. *r*=-0.620, *p* = 0.002, *N* = 22). These findings reveal the RSCP-elevated voiding flow was attributed mainly to a shortened voiding time but trivially to a modified voided volume.

### Lessened voiding workload

**Fig. 4:**
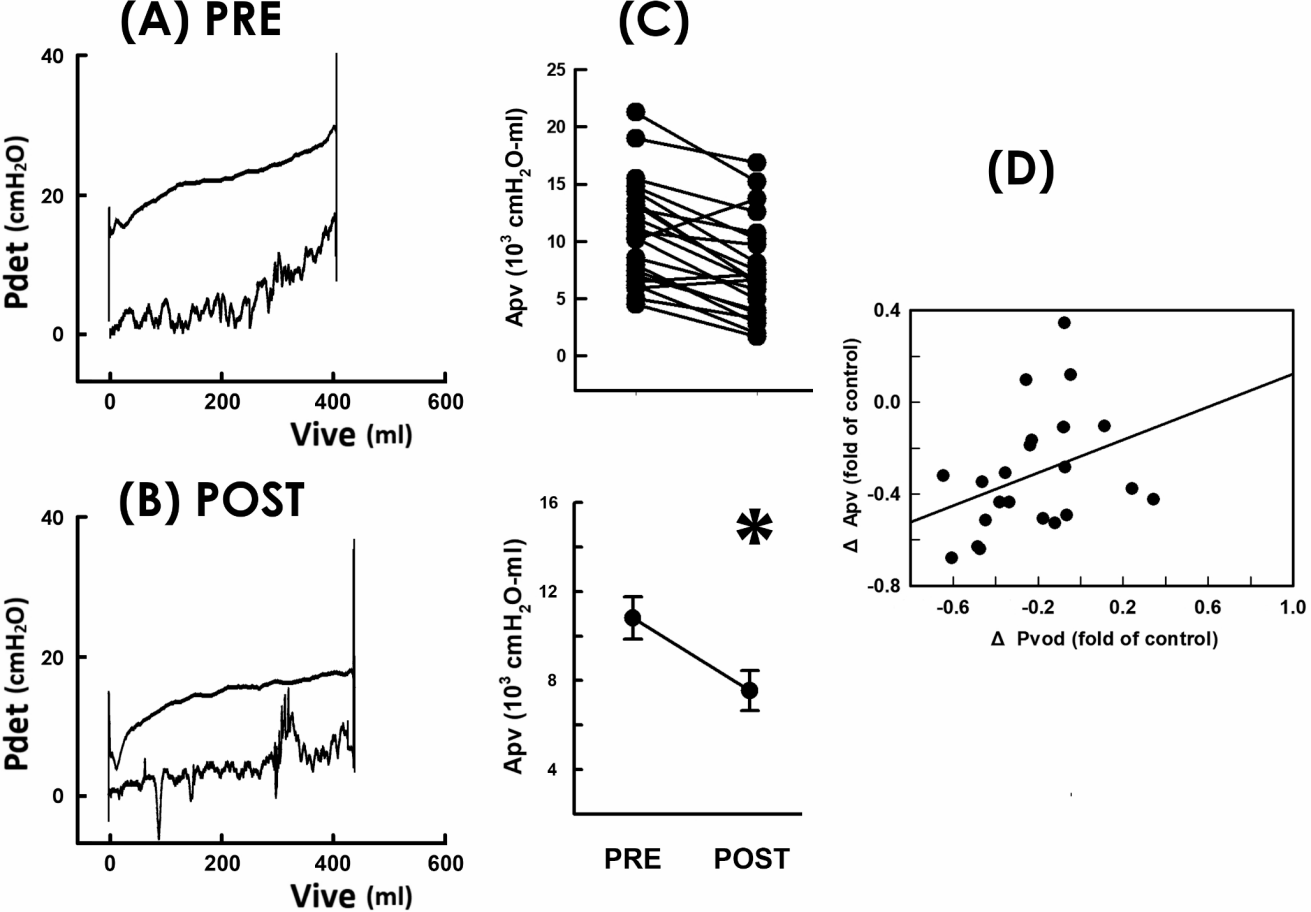
Pressure-volume analyses before and after RSCP. **A** and **B** Pressure-volume loops of a POP patient measured pre- and post-operatively, respectively (PRE and POST). Pdet: detrusor pressure, Vive: intra-vesical volume. **C** Individual (upper) and mean (lower) values of the loop-enclosed area (Apv) of the patient (* *p* = 0.017 vs. PRE; *N* = 22). **D** Correlation analysis of the RSCP-associated change in Apv (ΔApv) with the change in *mean voiding pressure (ΔPvod*; *r* = 0.385, *p* = 0.047; *N* = 22)

Having observed RSCP brought about an enhanced emission rate with a reduced pressure developed in POP patients, we wonder if these findings together imply RSCP lessened the workload of voiding. We thereby assayed the trajectory-enclosed area (Apv) of the pressure-volume analysis (PVA), because it is presumed to represent the work expenditure of the bladder in a voiding cycle [15–17]. Adapted from the time-domain cystometry (Fig. 1A and B), the pre- and post-operative PVAs were constructed by plotting Pdet in respect to Vive (Fig. 4A PRE and 4B POST). The trajectory of pressure-volume data moved counterclockwise and shaped an enclosed loop that signified a voiding cycle. When compared with the pre-operative control, RSCP decreased Apv as it distinctly depressed the top border of the loop without obviously affecting the left, right, and bottom borders. The Apv decrement was confirmed by summary data demonstrating RSCP post-operatively decreased Apv in most patients (Fig. 4C.19/22) and significantly decreased the mean Apv of the patient (*p* = 0.017 vs. PRE, *N* = 22) compared to the pre-operative control. Because Apv is an integral of pressure with volume; and the above results demonstrated RSCP decreased Pvod without obviously affecting Vvod, we next analyzed the correlation between the RSCP-associated changes in Apv and Pvod (ΔApv and ΔPvod, respectively) to elucidate their relationship. The bivariate analysis demonstrated ΔApv displayed a weak but significant correlation with the ΔPvod (Fig. 4D, *r* = 0.385, *p* = 0.047, *N* = 22), indicating the RSCP-decreased Apv was associated with the reduced Pvod.

## Discussion

### Advantage of PVA

In accompanied by a cystometry, the current study analyzed workload of voiding using PVA because lasting overload on the bladder could lead to un-compensatory voiding dysfunctions [24]. Cystometry is well established and widely used in laboratory investigations [13] and clinical scenarios [14] to evaluate bladder physiology and identify deficits of bladder function. Nevertheless, when assessing thermodynamic processes/performance of the bladder, cystometry has hereditary limitations as it off-line analyzes voiding workload that needs waiting for processing after the laboratory investigation. Moreover, the thermodynamic work of a voiding cannot be comprehended immediately using cystometry, rather it needs adroit calculation by specialists. Particularly, because the duration of urine emission in a time-domain cystometry is a relatively short period in the whole voiding cycle that results in a miserable resolution in changes of the pressure and volume during emission, thereby requires careful data acquisition and analysis by experts in urodynamics.

Notably, results in this study demonstrated PVA provides continuous and graphic monitoring of voiding dynamics. In addition, the trajectory loop in PVA offers a conceptual illustration of voiding work that can be easily comprehended and calculated by scientists/physicians with minimal processing. Moreover, the trajectory of urine emission was about a quarter of a loop in the PVA, thereby it offers a satisfactory resolution in the pressure-volume relationship during emission that can be clearly visualized and analyzed with negligible training in data acquisition/analysis.

Nowadays, as the advancements in computer technology, to simultaneously display cystometry with PVA is no longer a challenge. Therefore, investigations comprise PVA and an ongoing cystometry would provide on-line, continuous, and clear assessment of voiding thermodynamics that can be immediately and easily acquired with minimal processing and calculation.

### RSCP as a surgical option

Because restoration of apical support is critical for the surgical reconstruction of symptomatic POP, a laparotomy abdominal sacrocolpopexy with mesh was first established [7]; and then laparoscopic sacrocolpopexy was described [29]. Though the laparoscopic procedure achieves comparable therapeutic outcomes to abdominal sacrocolpopexy but with diminished blood loss and shortened hospital stay [30], it entails surgeons with good laparoscopic skills and elevated surgeon’s ergonomic strain [30]. Remarkably, since the US Food and Drug Administration approved the robotic system in 2005 [6], robotic-assisted gynecological surgery has markedly shortened the learning curve [8] and reduced surgeon’s ergonomics [7] compared with the conventional laparoscopy. Therefore, RSCP has gained popularity for the POP repair [4].

### Post-operative functional improvements

Emerging publications support benefits of RSCP to POP patients by showing RSCP results in satisfactory structural restoration [10] and improves quality of life subjectively [9]. Because to objectively evaluate effects of RSCP on bladder functions is crucial for clinicians when making a therapeutic decision; and functional improvement is one of the goals of POP repair [9]; moreover, considering bladder function involves adequate urine storage and efficient disposal [12], and a previous study has demonstrated RSCP ameliorates storage dysfunction in POP patients [11], the current study investigated the potential impact of RSCP on the voiding function of patients with POP.

By specifically lying focus on the resistance and resistance-dependent voiding workload, results in the current study demonstrated RSCP diminished the voiding resistance; and we suggest this effect could be attributed to that RSCP relieved the kinking and/or compression of the bladder outlet by restoring the anatomical confines of prolapsed organs [26]. Furthermore, given the voiding resistance is defined by dividing the voiding pressure by voiding flow, we specified the impact of RSCP on voiding pressure and flow. Results in this study demonstrated that associated with the diminished voiding resistance, RSCP consistently and significantly decreased voiding pressure and increase flow rate of the voiding. Together with bivariate analyses revealed the RSCP-diminished resistance correlated with both the changes in voiding pressure and voiding flow, these findings collectively support that the RSCP-diminished voiding resistance brought about depressed voiding pressure associated with an enhanced flow rate during voiding. We thereby propose the RSCP-diminished voiding resistance on one hand reduced bladder pressure developed for driving flow thereby lessened the voiding work; and on the other hand, prompted urine emission hence increased voiding efficacy.

### Relieved voiding workload

Our speculation is supported by lines of evidence. Firstly, analogously to the cystometry showing RSCP decreased voiding pressure without markedly affecting the voided volume, PVA demonstrated RSCP distinctly depressed the level of the top border (representing the voiding pressure) of the trajectory loop but negligibly affected the intercept between the right and the left borders (representing the voided volume) and the level of the bottom border (representing the baseline bladder pressure). Because the Apv is an integral of the pressure with respect to volume, these findings revealed the RSCP-decreased Apv was largely attributed to the decreased voiding pressure. This suggestion was supported by the finding that the RSCP-induced decrement in Apv was correlated with changes in voiding pressure. Given Apv is assumed to represent the thermodynamic work performed by the bladder in a voiding cycle [15–17], these results suggest the RSCP-diminished voiding resistance brought about decreased pressure developed for driving urine flow, and thereby lessened the voiding workload.

### Enhanced voiding efficacy

On the other hand, while the bladder post-operatively developed a decreased pressure, it drove urine flow with a higher emission rate that is evidenced by the cystometry showed the voiding flow was consistently and significantly increased after RSCP. Notably, though RSCP post-operatively displayed a trivial effect on the voided volume, it shortened the voiding time. Because the voiding flow is defined by dividing voided volume by voiding time; and the RSCP-induced flow increment correlated with the decrement in voiding time, these results not only support that RSCP post-operatively increased the emission rate but also reveal it enhanced voiding efficacy because bladder voided unmodified volume with a shortened time.

Together with above findings, these results collectively suggest RSCP diminished voiding resistance of the bladder that on one hand reduced the pressure gradient developed for driving urine flow that lessened the workload; and on the other hand, prompted urine emission that brings about an increased voiding efficacy.

### Limitations in this study

*Findings in this study have hereditary limitations in internal and external validity owing to a retrospective design. In addition*, *the patient number is not very large*,* thereby*,* the potential bias in the effects of measurement waits to be excluded using study with more patients.* Moreover, post-operative effects were measured 107.31 ± 11.10 days after the RSCP in this study. Given benefits of a surgery need to be monitored for a long period, a lasting follow-up of therapeutic outcome is needed to confirm the advantage of RSCP to POP patients.

## Conclusion

In summary, results in this study reveal RSCP diminished voiding resistance in POP patients that on one hand increased voiding efficacy by prompting urine emission; and on the other hand, lessened voiding workload via reducing the developed pressure for driving urine flow.
